# Supplementary material for: Identification of Structural Variation from NGS-Based Non-Invasive Prenatal Testing
Source: Int J Mol Sci. 2019 Sep 7;20(18):4403. doi: 10.3390/ijms20184403 (PMC6769840; doi:10.3390/ijms20184403)
Supplement: Supplementary file 1 [file ijms-20-04403-s001.zip › Caption - Supplementary Files.pdf]

Supplementary Figure 1: Graphical representation of maternal CNVs identified from the NIPT compared to ClinVar database results. Identified CNVs (Light blue), GC content (Red), Unmappable regions (Grey), ClinVar benign and likely benign CNVs (Green), ClinVar pathogenic and likely pathogenic CNVs (Orange).

Supplementary Table 1: List of maternal CNVs identified from the NIPT.
