# Supplementary material for: Identification of Structural Variation from NGS-Based Non-Invasive Prenatal Testing
Source: Int J Mol Sci. 2019 Sep 7;20(18):4403. doi: 10.3390/ijms20184403 (PMC6769840; doi:10.3390/ijms20184403)
Supplement: Supplementary file 1 [file Supplementary Figure 1.pdf › Supplementary Figure 1.pdf]

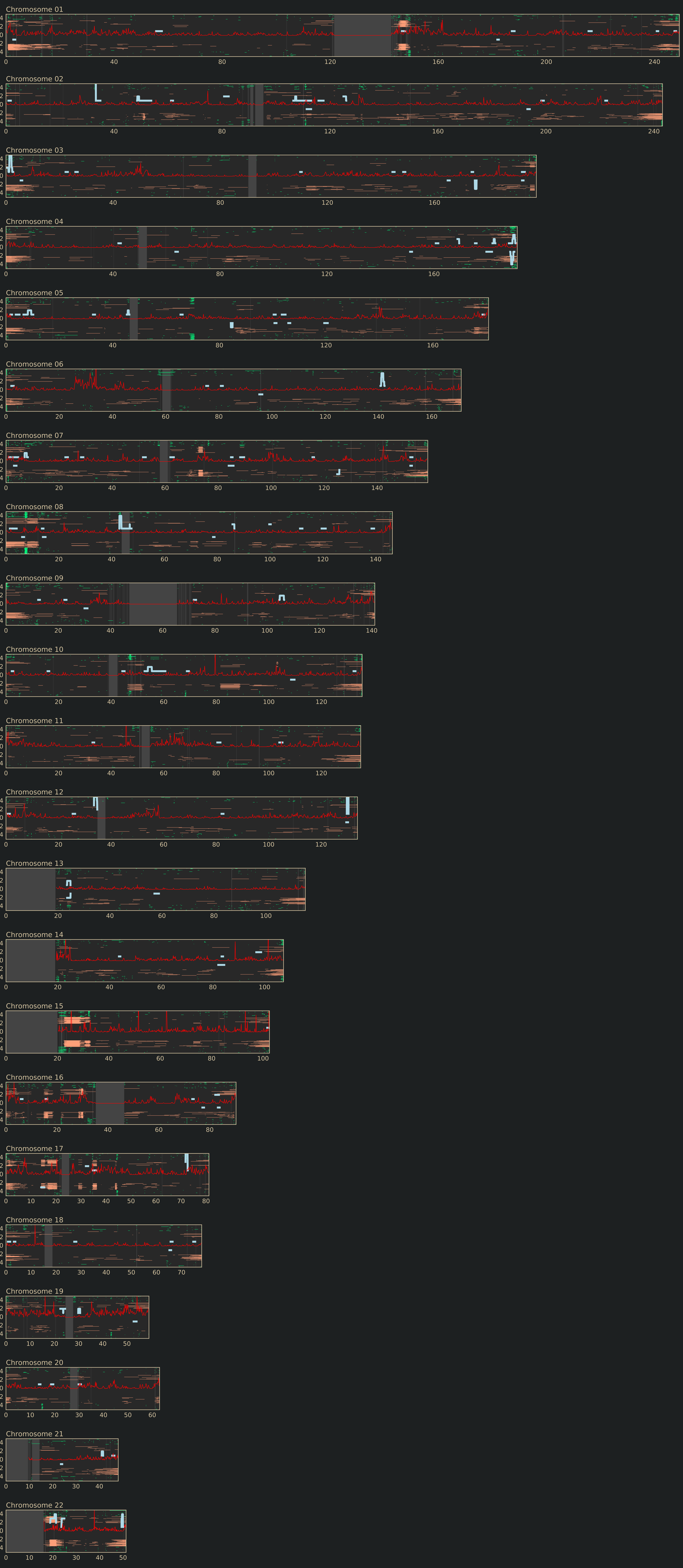
Chromosome 01
Chromosome 02
Chromosome 03
Chromosome 04
Chromosome 05
Chromosome 06
Chromosome 07
Chromosome 08
Chromosome 09
Chromosome 10
Chromosome 11
Chromosome 12
Chromosome 13
Chromosome 14
Chromosome 15
Chromosome 16
Chromosome 17
Chromosome 18
Chromosome 19
Chromosome 20
Chromosome 21
Chromosome 22
